# Screening for Anemia and Iron Deficiency in the Adult Portuguese Population

**DOI:** 10.1155/2020/1048283

**Published:** 2020-07-29

**Authors:** António Robalo Nunes, João Mairos, Dialina Brilhante, Filipa Marques, Aurora Belo, José Cortez, Cândida Fonseca

**Affiliations:** ^1^Anemia Working Group Portugal, Av. Columbano Bordalo Pinheiro 75, 1070-061 Lisbon, Portugal; ^2^Immunohemotherapy Service, Armed Forces Hospital (HFAR)–Lisbon, Azinhaga Dos Ulmeiros, Paço Do Lumiar, 1690-020 Lisbon, Portugal; ^3^Air Force Health Directorate–Lisbon, Azinhaga Dos Ulmeiros, Paço Do Lumiar, 1690-020 Lisbon, Portugal; ^4^Immunohemotherapy Service, Portuguese Institute of Oncology of Lisbon Francisco Gentil (IPO Lisboa), R. Prof. Lima Basto, 1099-023 Lisbon, Portugal; ^5^Medicine Service, São Francisco Xavier Hospital, Western Lisbon Hospital Center (CHLO), Estrada Do Forte Do Alto Do Duque, 1449-005 Lisbon, Portugal; ^6^Clinical Medicine Department, Armed Forces Hospital (HFAR)–Lisbon, Azinhaga Dos Ulmeiros, Paço Do Lumiar, 1690-020 Lisbon, Portugal; ^7^Clinical Pathology Department, Portuguese Institute of Oncology of Lisbon Francisco Gentil (IPO Lisboa), R. Prof. Lima Basto, 1099-023 Lisbon, Portugal; ^8^Nova Medical School, Faculty of Medical Sciences, Campo Mártires da Pátria 130, 1169-056 Lisbon, Portugal

## Abstract

Anemia and iron deficiency (ID) can impair quality of life and socioeconomic development. We evaluated the prevalence of anemia and ID in the adult Portuguese population in real-life contexts by gender, age, and pregnancy status. We performed a cross-sectional screening in adult individuals in mainland Portugal from 2013 to 2017. Participants completed a survey about demographics and signs or symptoms compatible with anemia, and ID and hemoglobin and ferritin concentrations were determined by point-of-care tests. We estimated and compared prevalence ratios (PR) of anemia and ID using Poisson regression with robust variance and the Wald chi-square test. We collected data from 11,030 individuals (26% men, 64% nonpregnant women, and 10% pregnant women). We found anemia in 51.8% (95% CI 50.1–53.4%) of nonpregnant women in fertile age, 46.6% (95% CI 44.7–48.6%) of nonpregnant women >51 years, 38.2% (95% CI 35.4–41.1%) of pregnant women, and 33.3% (95% CI 31.6–35.1%) of men. The prevalence of ID was 72.9% (95% CI 71.4–74.4%) in nonpregnant women in fertile age, 50.5% (95% CI 48.5–52.4%) in nonpregnant women >51 years, 94.8% (95% CI 93.3–96.0%) in pregnant women, and 28.9% (95% CI 27.3–30.6%) in men. We found significant associations between the prevalence of anemia or ID and nonpregnant women (PR: 1.50, 95% CI 1.42–1.59 or PR: 2.21, 95% CI 2.09–2.35, respectively), manifestation of signs or symptoms (PR: 1.19, 95% CI 1.53–1.23 or PR: 1.22, 95% CI 1.18–1.26), pregnant women (PR: 0.74, 95% CI 0.68–0.80 or PR: 1.30, 95% CI 1.27–1.33), and nonpregnant women ≤51 years (PR: 1.11, 95% CI 1.06–1.17 or PR: 1.42, 95% CI 1.36–1.48). In conclusion, anemia and ID represent moderate to severe public health problems, particularly among women in fertile age and in 3rd trimester, of pregnancy emphasizing the need to raise the public and health professionals' awareness of these problems and their prevention, diagnosis, and treatment.

## 1. Introduction

Anemia, defined as a decreased hemoglobin concentration [1], is a major global public health problem affecting about one-quarter of the world's population [2]. In Europe, the World Health Organization (WHO) has estimated a prevalence of anemia of approximately 23% for children ≤5 years, 23% for nonpregnant women in fertile age, and 26% for pregnant women in 2011 [3]. In Portugal, recent studies have estimated a prevalence of anemia of 20% in the Portuguese general population, particularly affecting women (21%), pregnant women (54%), and adults aged ≥65 years (21%) [4, 5].

Anemia may result from several causes, such as micronutrient deficiencies (iron, folate, and vitamin B12), genetic disorders, or other conditions that may induce iron loss or decreased iron absorption (acute or chronic infection, inflammatory bowel disease, chronic heart failure, chronic kidney disease, neoplasm, and autoimmune disease) [3, 6, 7]. Iron deficiency (ID) is a major contributing factor of anemia in developed countries, generally caused by an insufficient dietary iron intake or by conditions causing hemorrhage or decreased iron absorption [3, 6–8]. Approximately, 50% of anemia cases can be explained by ID. However, this proportion can vary among different local conditions and population groups [3–5, 9–12]. ID is more likely to occur when the iron requirements are increased, such as during periods of rapid growth and increased erythropoiesis (children and adolescents), additional requirements (pregnancy), or due to menstrual bleeding and insufficient dietary iron intake (women in fertile age) [8, 11–13]. In contrast, the elderly population is particularly susceptible to anemia of chronic disease, which generally lacks a known underlying cause [6, 10]. Anemia of chronic disease is commonly associated with several prevalent conditions in the elderly population, such as chronic infections, inflammatory diseases (heart failure, chronic kidney disease, and immune diseases), and neoplasms [10]. Common causes of ID in the elderly population include disorders and/or acute or chronic hemorrhage through the gastrointestinal tract [8, 10].

Anemia and ID can have adverse health effects impairing quality of life [14, 15] and socioeconomic development [3, 16]. In children, ID anemia negatively affects motor and cognitive development, and, in adults, it is associated with fatigue, decreased physical performance, and lower productivity [3, 8, 12]. During pregnancy, ID anemia has been associated with low birth weight, premature delivery, and increased risk of perinatal mortality [3, 12, 13]. In the elderly population, anemia is associated with frailty, decreased cognitive function, and a global increase in morbidity and mortality [10]. ID, by itself, can have deleterious effects as the use of iron supplementation to correct ID without anemia has been associated with beneficial effects in women in fertile age or pregnancy [8].

The high prevalence and negative impact of anemia and ID in high-risk population groups make these conditions public health problems that must be taken into consideration [3–5, 7, 10]. However, the relevance and best strategies of screening and treating ID or ID anemia in different populations are still unclear [6, 8]. On the one hand, ID is a widespread problem in clinical practice, and several guidelines for the diagnosis and treatment of anemia or ID exist and vary between different medical specialties and populations [6, 7]. On the other hand, to increase the prevention or treatment of anemia and ID, additional studies are needed to characterize the local etiology, prevalence, and most affected population groups [3, 17]. Finally, epidemiological data on anemia and ID are sparse [4, 5, 9, 11, 18]. Therefore, we aimed to evaluate the prevalence of anemia and ID in different groups of the adult Portuguese population stratified by age, gender, and in the case of women, by pregnancy status. This study was conducted in real-life contexts, in which the participants were asked to complete a survey as well as screening tests for anemia and ID. Additionally, we aimed to raise awareness among the public, health professionals, and policy-makers about the extent of anemia and ID problem in Portugal and possible future interventions.

## 2. Materials and Methods

### 2.1. Study Design

This cross-sectional screening was promoted by the Anemia Working Group Portugal—*Associação Portuguesa para o Estudo da Anemia*—aiming to evaluate the prevalence of anemia and ID in different demographic groups of the adult Portuguese population. The screening was performed using a convenience sampling in real-life contexts, such as public locations and private entities frequented by the general population. The screening was carried out in several geographical locations in mainland Portugal from January 2013 to December 2017. The study was conducted by applying a survey as well as anemia and ID screening blood tests to the participants, in which hemoglobin and ferritin concentrations were determined by point-of-care tests.

The study received approval from the Portuguese National Data Protection Committee (CNPD, Lisbon, Portugal). All the participants voluntarily participated in this study and provided their oral consent after clarification about the scope of the screening.

### 2.2. Participants and Procedures

The study enrolled all adult individuals (≥18 years old) that, after an invitation to participate in the screening in several public locations, such as public or private health institutions, pharmacies, shopping centers, companies, and medical and healthcare congresses, they showed availability and interest to participate.

The participants were asked to answer a survey about anemia and ID. The survey included questions about demographic characteristics and the presence or absence of signs or symptoms compatible with the presence of anemia or ID. The last were collected using three categories: (1) fatigue in daily activities; (2) bleed easily, headaches, and dizziness; and (3) visible blood loss. If the participants answered positively to at least one question of these categories, they were considered as having a sign or symptom compatible with the presence of anemia or ID (see Figure S1 in the Supplementary Material for analysis of the survey's form).

Hemoglobin and ferritin concentrations were determined by point-of-care testing devices using capillary puncture performed by the research team. This team consisted of trained individuals in the survey methodology and the execution of point-of-care tests by capillary puncture, mostly health professionals (nurses and lab technicians). Hemoglobin and ferritin concentrations were determined using Cera-Chek Hb Plus (Ceragem Medisys, Chungnam, South Korea) and Vedalab Easy Reader (Vedalab, Alençon, France), respectively.

### 2.3. Definition of Anemia and Iron Deficiency

Anemia was defined as hemoglobin concentration <12.0 g/dL for nonpregnant women, <13.0 g/dL for men, <11.0 g/dL for pregnant women in the 1st or 3rd trimester, and <10.5 g/dL for pregnant women in the 2nd trimester, in accordance with the WHO [1] and the Portuguese Directorate-General for Health (DGS, Lisbon, Portugal) [19] guidelines.

ID was classified according to the ferritin concentration, which is positively correlated with the magnitude of the total body iron stores in the absence of inflammation [20]. Several ferritin cutoffs have been proposed in the literature for the diagnosis of ID in the general population, and there are no consensus criteria [7]. Ferritin concentrations <15 ng/mL are indicative of iron stores depletion in both genders [20]. In the presence of factors that affect ferritin levels such as age, inflammation, infection, or pregnancy, ferritin concentrations <30 ng/mL are also indicative of iron stores depletion [20]. The DGS indicates serum ferritin concentrations of 30 to 340 ng/mL as normal reference ranges in the general adult population and recommends that pregnant women should initiate oral iron therapy if their serum ferritin concentration is <70 ng/mL [19]. Therefore, in this study, ID was defined as a ferritin concentration <30 ng/mL in men and nonpregnant women and <70 ng/mL in pregnant women, regardless of the pregnancy trimester [19].

### 2.4. Statistical Analysis

A convenience sampling of the general adult population was conducted at several public and private locations for five consecutive years in mainland Portugal. A sample size calculation was not performed. Participants were excluded from the analysis if they had missing data regarding any of the following variables: age, gender, hemoglobin concentration, ferritin concentration, or pregnancy trimester.

Data from the sample population were stratified by gender, age, and presence or absence of signs or symptoms of anemia or ID, to estimate and compare the prevalence of anemia or ID between groups. Female participants were stratified into nonpregnant women (age ≥18 years) and pregnant women in the 1st, 2nd, or 3rd trimester of gestation. Nonpregnant women in fertile age (18–44 years) and pregnant women were matched by the following age groups: 18–26, 27–35, and 36–44 years. To evaluate the relationship of menopause with the prevalence of anemia or ID, nonpregnant women were further stratified into the age groups ≤51 years and >51 years. Because the scope of the screening did not include the report of a clinical diagnosis of menopause (i.e., 12 consecutive months of amenorrhea without any other obvious pathological or physiological cause) and because menopause is an event that occurs at a median age of 51 years [21–24], we have used this cutoff to stratify women into pre- and postmenopause.

The normality of the data was assessed using the Kolmogorov–Smirnov test. Continuous variables were presented as mean (standard deviation), median (minimum–maximum), or median (1st quartile–3rd quartile), as applicable. Categorical variables were expressed as number and percentage. Each prevalence estimate of anemia or ID was expressed as a percentage with the respective 95% Clopper–Pearson confidence interval (CI). To assess the relationship between anemia or ID and different population groups or the presence of signs or symptoms of anemia or ID, crude prevalence ratios (PR) with their corresponding 95% CI were estimated using Poisson regression with robust variance. Pairwise comparisons of each PR versus the reference group were performed using the Wald chi-square test.

Statistical significance was reported for *p* value <0.05. All data analyses were carried out using SPSS for Windows, version 25.0 (SPSS Inc., Chicago, IL, USA).

## 3. Results

### 3.1. Sample Characterization

During the study period, between 2013 and 2017, we collected data from 11,384 participants that resulted from 135 events of anemia and ID screenings. We excluded 354 cases from the initial screening sample, corresponding to participants with age <18 years (*n* = 86) or participants' records that presented missing data (*n* = 268).

We included 11,030 adult participants with ages between 18 and 99 years, 25.8% (*n* = 2845) of men, 64.0% (*n* = 7060) of nonpregnant women, and 10.2% (*n* = 1125) of pregnant women. Their mean age was 40.90 (16.86), 45.94 (16.02), and 30.89 (4.84), respectively.  Table 1 shows the summary statistics for demographic characteristics and hemoglobin and ferritin concentrations of the study participants.

### 3.2. Prevalence of Anemia and Iron Deficiency

#### 3.2.1. Health Institutions versus Other Public Locations

The prevalence and prevalence ratios of anemia and ID by the type of location of the participant are presented in Supplementary Table S2 (Supplementary Materials). We estimated an anemia prevalence of 56.7% (95% CI 55.2–58.1%) in participants from health institutions and 35.6% (95% CI 34.5–36.8%) in participants from other public locations. Furthermore, we found a significant association between having anemia and the type of institution of the participant. Compared with participants from other public locations, participants from health institutions were associated with a PR of anemia of 1.59 (95% CI 1.50–1.68, *p* < 0.001).

Regarding ID, we estimated a prevalence of 59.6% (95% CI 58.2–61.0%) in participants from health institutions and 57.0% (95% CI 55.8–58.3%) in participants from other public locations. No significant association was found between the prevalence of ID and the type of institution of the participant.

#### 3.2.2. Men versus Nonpregnant Women

**Figure 1:**
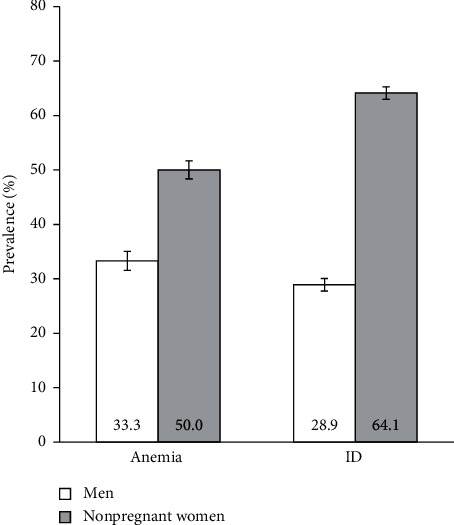
Prevalence of anemia and iron deficiency in men (white bars, *n* = 2845) and nonpregnant women (grey bars, *n* = 7060). Error bars indicate 95% confidence intervals. ID, iron deficiency.

The prevalence of anemia and ID by gender is presented in Figure 1. We estimated an anemia prevalence of 33.3% (95% CI 31.6–35.1%) in men and 50.0% (95% CI 48.9–51.2%) in nonpregnant women. Furthermore, we found a significant association between having anemia and gender. Compared with men, nonpregnant women were associated with a PR of anemia of 1.50 (95% CI 1.42–1.59, *p* < 0.001).

Regarding ID, we estimated a prevalence of 64.1% (95% CI 62.9–65.2%) in nonpregnant women and 28.9% (95% CI 27.3–30.6%) in men. Similar to anemia, nonpregnant women were associated with a significantly higher prevalence of ID compared with men (PR: 2.21, 95% CI 2.09–2.35; *p* < 0.001).

#### 3.2.3. Presence of Signs or Symptoms of Anemia or Iron Deficiency

**Figure 2:**
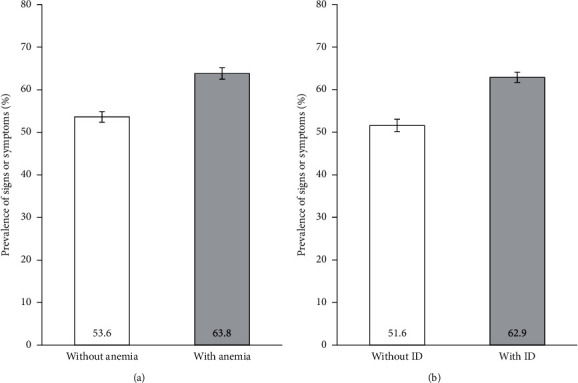
Prevalence of the presence of signs or symptoms of anemia or iron deficiency in (a) participants without anemia (*n* = 6120) or with anemia (*n* = 4910) and (b) participants without iron deficiency (*n* = 4619) or with iron deficiency (*n* = 6411). Error bars indicate 95% confidence intervals. ID, iron deficiency.

Figure 2 shows the prevalence of signs or symptoms compatible with anemia or ID by the anemia or ID status in the study sample. Most of the participants with anemia (63.8%, 95% CI 62.4–65.1%) or with ID (62.9%, 95% CI 61.7–64.1%) presented signs or symptoms compatible with these conditions. Furthermore, we found a statistically significant association between the presence of signs or symptoms and the presence of anemia or ID. Participants with anemia were associated with a PR of 1.19 (95% CI 1.15–1.23, *p* < 0.001) compared with those without anemia. Similarly, participants with ID had a statistically higher prevalence of signs or symptoms compared with participants without ID (PR: 1.22, 95% CI 1.18–1.26; *p* < 0.001).

#### 3.2.4. Nonpregnant Women versus Pregnant Women

Table 2 shows the prevalence and the PR of anemia in nonpregnant women in fertile age and pregnant women stratified by age and pregnancy trimester. Overall, we found a prevalence of anemia of 51.8% (95% CI 50.1–53.4%) in nonpregnant women in fertile age and 38.2% (95% CI 35.4–41.1%) in pregnant women. Furthermore, we estimated a significant association between anemia and pregnancy status, in which pregnant women had a 0.74-fold lower prevalence of anemia compared to nonpregnant women in fertile age (Table 2). This significant lower PR of anemia in pregnant women versus nonpregnant women in fertile age was observed in all age groups (18–26 years, 27–35 years, and 36–44 years).

When analyzing pregnant women by their pregnancy trimester, we recorded that almost half of the pregnant women in the 3rd trimester (48.6%, 95% CI 43.9–53.3%) had anemia compared to less than one-third of the pregnant women in the 1st (26.8%, 95% CI 14.2–42.9%) and 2nd trimesters (31.6%, 95% CI 28.0–35.4%). What stands out in Table 2 is the high prevalence of anemia in 3rd trimester pregnant women, which was not significantly different compared to nonpregnant women in fertile age in all groups. Conversely, pregnant women in the 1st and 2nd trimesters had a significant 48% and 39% decrease, respectively, in the overall prevalence of anemia compared to nonpregnant women in fertile age (Table 2).

Table 3 shows the prevalence and the PR of ID in nonpregnant women in fertile age (ferritin <30 ng/mL) and in pregnant women (ferritin <70 ng/mL) stratified by age and pregnancy trimester. In the total group, the prevalence of ID was 72.9% (95% CI 71.4–74.4%) in nonpregnant women in fertile age and 94.8% (95% CI 93.3–96.0%) in pregnant women. The most striking result to emerge from the data is that pregnant women in the 2nd and 3rd trimesters presented a prevalence of ID above 90%, regardless of the age group. The 3rd trimester pregnant women showed the highest prevalence, ranging from 97.8% (95% CI 92.1–99.7%) in the 36–44-year group to 98.7% (95% CI 92.9–100) in the 18–26-year group.

Moreover, we found that pregnant women had a prevalence of ID that was 1.30-fold higher than nonpregnant women in fertile age in the total group (Table 3), suggesting that ID was associated with the pregnancy status. We found the highest PR for ID in 3rd trimester pregnant women, compared to nonpregnant women in fertile age in the total group (PR: 1.34, 95% CI 1.31–1.38; *p* < 0.001) and the 36–44-year group (PR: 1.40, 95% CI 1.33–1.46; *p* < 0.001).

**Figure 3:**
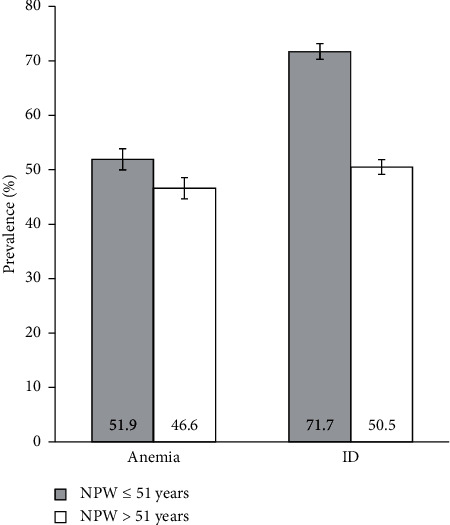
Prevalence of anemia and iron deficiency in nonpregnant women aged ≤51 years (grey bars, *n* = 4525) and >51 years (white bars, *n* = 2535). Error bars indicate 95% confidence intervals. ID, iron deficiency; NPW, nonpregnant women.

We also aimed to compare the prevalence of anemia or ID between nonpregnant women in pre- and postmenopause, based on the stratification of nonpregnant women into the age groups ≤51 and >51 years.  Figure 3 shows the prevalence of anemia and ID in these groups of nonpregnant women.

We found a prevalence of anemia of 51.9% (95% CI 50.5–53.4%) in nonpregnant women ≤51 years and 46.6% (95% CI 44.7–48.6%) in nonpregnant women >51 years. Moreover, we found a significant association between these age groups and the prevalence of anemia, which showed an 11% increase in nonpregnant women ≤51 years compared to nonpregnant women >51 years (PR: 1.11, 95% CI 1.06–1.17; *p* < 0.001).

We found similar results for ID. The prevalence of ID was 71.7% (95% CI 70.3–73.0%) in nonpregnant women ≤51 years and 50.5% (95% CI 48.5–52.4%) in nonpregnant women >51 years. We also found a significant association between these age groups and prevalence of ID, which showed a 42% increase in nonpregnant women ≤51 years compared to nonpregnant women >51 years (PR: 1.42, 95% CI 1.36–1.48; *p* < 0.001). The results and discussion may be presented separately, or in one combined section, and may optionally be divided into headed subsections.

## 4. Discussion

In this study, we conducted a large-scale screening (*n* = 11030) for anemia and ID in the adult Portuguese population between 2013 and 2017. This study was performed in real-life contexts both within and outside the clinical setting, allowing not only to obtain more prevalence data on population groups generally less accessible outside the clinical context, such as adult men, but also on high-risk groups within the population, such as pregnant women. Therefore, we analyzed the extent of anemia and ID, which has been poorly studied in different population groups, to increase the amount of epidemiological data available for healthcare planning.

We found anemia to be highly prevalent in the screened adult general population: 33% in men, 38% in pregnant women, 47% in nonpregnant women >51 years, and 52% in both in nonpregnant women in fertile age (18–44 years) and nonpregnant women <51 years. These prevalence estimates of anemia are above the ones previously reported in the WHO [3] and EMPIRE [4, 5] studies on the Portuguese general population. The prevalence of anemia was particularly high in nonpregnant women in fertile age, exceeding the estimated value for pregnant women, therefore contrasting with the estimates from WHO (19% nonpregnant versus 26% pregnant) [3] and the EMPIRE study (21% nonpregnant versus 54% pregnant) [4]. These differences may be explained not only by different study designs but also by differences in the demographic characteristics and the sample size used to estimate the prevalence of anemia. In the present study, we analyzed 3500 nonpregnant women in fertile age and 1125 pregnant women, whereas the EMPIRE study [4] analyzed 2245 and 59, respectively. Nevertheless, our findings are consistent with that of the EMPIRE study [4] because the reported prevalence estimates make anemia a moderate (20–39%) to severe (≥40%) public health problem in the population groups at higher risk, namely, the women in fertile age both nonpregnant or pregnant [2, 16].

In this study, we have reported a higher prevalence of cases with signs or symptoms compatible with anemia or ID in participants with anemia or with ID. Although the presence of anemia or ID has shown a significant association with the manifestation of compatible signs or symptoms, these are generally nonspecific and can result from several etiologies and comorbidities [6].

The ID was also found to be highly prevalent in the screened adult general population. Adult men showed the lowest prevalence of ID (29%), whereas nonpregnant women >51 years, nonpregnant women in fertile age, and pregnant women presented a prevalence of ID of 51%, 72%, and 95%, respectively. Similar to anemia, the prevalence estimates for ID are above those previously reported by the EMPIRE study [4], mainly for nonpregnant women in fertile age (38%) and pregnant women (63%). It should be highlighted that, despite being in line with the EMPIRE study, our results support a higher prevalence of both anemia and ID, which could be explained by the different study designs, as already mentioned, and also by the increased number of women participants in our study, including nonpregnant women of fertile age (*n* = 3500) and ≤51 years (*n* = 4525), as well as pregnant women (*n* = 1125).

We found significant associations between gender and the prevalence of anemia and ID when we compared men to nonpregnant women. When analyzing the proportion of cases with ID and anemia, we observed a prevalence of ID of 29% and a prevalence of anemia of 33% in men, and a prevalence of ID of 64% and prevalence of anemia of 50% in nonpregnant women. Because ID is the most common cause of anemia [3, 6], an increase in ID is expected to be accompanied by an increase in anemia [16]. On the contrary, depending on its etiology, ID includes both iron depletion stages without manifestation of anemia and more severe stages with progression to ID anemia [6]. In theory, if a given population presents a prevalence of ID anemia over 20%, a prevalence of some degree of ID around 50% is expected. If the prevalence of ID anemia exceeds 40%, then almost all population will present some degree of ID [16]. However, in this study, we estimated the overall prevalence of anemia, without further investigation of its underlying cause or the concomitant presence or absence of an ID, which limits the interpretations of the relative proportions of anemia and ID in men and nonpregnant women [16].

Nonpregnant women in fertile age presented a significantly increased prevalence of anemia compared to pregnant women, being this association significant. This finding may be explained by the increased prevention or surveillance of pregnant women in Portugal, being this population group usually targeted for additional clinical follow-up. Nonetheless, despite being normally followed early during pregnancy, iron deficiency is still underdiagnosed in Obstetrics and General and Family Medicine, in which ferritin levels are not mandatorily assessed. Furthermore, the application of the DGS guideline for the approach, diagnosis, and treatment of ID in adults [19] recommends pregnant women to initiate oral iron supplementation only when their serum ferritin concentration is <70 ng/mL. On the contrary, the difference between the prevalence of anemia in nonpregnant women and pregnant women should be scrutinized considering the pregnancy trimester. For instance, this difference can be explained by the suppression of menstrual blood loss during the 1st pregnancy trimester. Globally, we found that pregnant women in the 3rd trimester, when a significant increase in iron demand occurs, present the highest prevalence of anemia and ferritin concentrations <70 ng/mL, whereas pregnant women in the 1st trimester presented a lower prevalence of these conditions. We also found significant associations between ID and pregnancy status, and the variation of anemia and ID prevalence along the pregnancy trimesters was as expected. In pregnant women, the ferritin concentration increases in the initial gestation period in the 1st trimester and tends to progressively decrease during 32 weeks to about 50% concerning the prepregnancy levels, due to hemodilution and iron mobilization [25]. Therefore, variations in the ferritin concentration are influenced by the progressive increase of iron requirements during the 2nd and 3rd pregnancy trimesters, induced by growth, fetal-placental and maternal tissue development, and expansion of maternal red blood cell mass [19, 26]. As this expansion increases during the 2nd half of the 2nd trimester, the iron requirements reach their highest level during the last six to eight pregnancy weeks [26].

As for the strengths of this study, we highlight its implementation over a long period in a population-based sample in real-life contexts, in several public locations visited by the general population, the use of validated analytical tests for the determination of anemia and ID and the sample size. However, this study had some limitations that are intrinsic to screening methodologies. We have used a convenience sampling that depended on participants who were willing to participate, and no sample size calculations were performed to estimate the representativeness of the population groups. Thus, despite the considerable sample size, caution is needed when generalizing the results of this study to the different population groups since they have different availabilities to participate. Furthermore, the validity of self-reported data regarding signs or symptoms of anemia or ID and medical history is influenced by the participants' memory and intellectual capacities. Finally, other factors contributing to the prevalence of anemia and ID were not analyzed [16], such as diet, clinical follow-up, comorbidities, and adherence to iron supplementation, mainly in pregnant women.

## 5. Conclusions

We previously estimated prevalence levels of anemia or ID above 20% in the adult Portuguese general population. In this study, our findings provided additional evidence on the existence of a moderate to severe public health problem, particularly among adult women in fertile age and pregnant women in the 3rd trimester, claiming for a preventive intervention concerning a clarification in timing, duration, and trigger cutoff.

This screening provided a real-life picture allowing to raise awareness among the public, healthcare professionals, and policy-makers about the need for early and proactive diagnosis of anemia and ID. The implementation of future policies should promote further awareness and prevention of anemia and ID among the high-risk population groups, an active demand for healthcare, and better treatment strategies, to minimize the anemia and ID problem in Portugal.

## Figures and Tables

**Table 1:** Summary of the demographic characteristics and concentrations of hemoglobin and ferritin of the study participants.

Group	Participants, *N* = 11,030			
	*n*	Age, mean (SD) (years)	Hb, mean (SD) (g/dL)	Ferritin, median (*Q*1–*Q*3) (ng/mL)
Men	2845	40.90 (16.86)	13.62 (1.65)	50.82 (26.60–86.80)
Nonpregnant women				
Total	7060	45.94 (16.02)	12.01 (1.54)	21.28 (9.00–40.46)
≤51 years	4525	36.02 (9.04)	11.94 (1.53)	17.78 (9.00–33.46)
>51 years	2535	63.66 (8.91)	12.14 (1.55)	29.96 (14.28–56.00)

Pregnant women				
Total	1125	30.89 (4.84)	11.15 (1.41)	17.38 (9.00–30.94)
1st trimester	41	30.44 (5.26)	11.74 (1.40)	29.60 (9.00–45.49)
2nd trimester	633	30.67 (4.75)	11.16 (1.39)	20.44 (9.00–36.12)
3rd trimester	451	31.24 (4.90)	11.09 (1.44)	10.80 (9.00–23.94)
18–26 years	203	23.90 (2.08)	11.23 (1.51)	16.80 (9.00–29.12)
27–35 years	723	30.87 (2.51)	11.13 (1.40)	17.78 (9.00–31.78)
36–44 years	199	38.07 (1.89)	11.14 (1.36)	16.94 (9.00–30.94)

Nonpregnant women in fertile age^a^				
Total	3500	32.54 (7.14)	11.96 (1.52)	16.94 (9.00–32.20)
18–26 years	870	23.24 (2.37)	11.95 (1.53)	14.10 (9.00–27.44)
27–35 years	1285	30.91 (2.61)	12.01 (1.47)	17.78 (9.00–32.62)
36–44 years	1345	40.13 (2.53)	11.92 (1.56)	17.78 (9.00–35.28)

Hb, hemoglobin; *N*, total number of participants; *n*, number of participants per group; *Q*1, 1st quartile; *Q*3, 3rd quartile; SD, standard deviation. ^a^Subgroups of nonpregnant women matched to the same age groups of the pregnant women.

**Table 2:** Prevalence and prevalence ratio of anemia in nonpregnant and pregnant women stratified by age group and pregnancy trimester.

Group	Anemia			
	*N*	*n*	Prevalence, % (95% CI)	PR (95% CI)
Total				
NPW in fertile age^a^	3500	1812	51.8 (50.1–53.4)	1 (reference)
Pregnant women	1125	430	38.2 (35.4–41.1)	0.74 (0.68–0.80)^*∗∗∗*^
1st trimester	41	11	26.8 (14.2–42.9)	0.52 (0.31–0.86)^*∗*^
2nd trimester	633	200	31.6 (28.0–35.4)	0.61 (0.54–0.69)^*∗∗∗*^
3rd trimester	451	219	48.6 (43.9–53.3)	0.94 (0.85–1.04)

18–26 years				
NPW in fertile age^a^	870	445	51.1 (47.8–54.5)	1 (reference)
Pregnant women	203	74	36.5 (29.8–43.5)	0.71 (0.59–0.86)^*∗∗*^
1st trimester	10	0	0.0 (0.0–30.8)	-^b^
2nd trimester	117	33	28.2 (20.3–37.3)	0.55 (0.41–0.74)^*∗∗∗*^
3rd trimester	76	41	53.9 (42.1–65.5)	1.06 (0.85–1.31)

27–35 years				
NPW in fertile age^a^	1285	634	49.3 (46.6–52.1)	1 (reference)
Pregnant women	723	281	38.9 (35.3–42.5)	0.79 (0.71–0.88)^*∗∗∗*^
1st trimester	23	10	43.5 (23.2–65.5)	0.88 (0.55–1.41)
2nd trimester	414	133	32.1 (27.6–36.9)	0.65 (0.56–0.76)^*∗∗∗*^
3rd trimester	286	138	48.3 (42.3–54.2)	0.98 (0.86–1.12)

36–44 years				
NPW in fertile age^a^	1345	733	54.5 (51.8–57.2)	1 (reference)
Pregnant women	199	75	37.7 (30.9–44.8)	0.69 (0.58–0.83)^*∗∗∗*^
1st trimester	8	1	12.5 (0.3–52.7)	0.23 (0.04–1.44)
2nd trimester	102	34	33.3 (24.3–43.4)	0.61 (0.46–0.81)^*∗∗*^
3rd trimester	89	40	44.9 (34.4–55.3)	0.83 (0.65–1.04)

CI, confidence interval; *N*, total number of participants; *n*, number of participants with anemia; NPW, nonpregnant women; PR, prevalence ratio. ^a^Subgroups of nonpregnant women matched to the same age groups of the pregnant women. ^b^Poisson regression was not performed because the prevalence equals zero. ^*∗*^*p* value <0.05, ^*∗∗*^*p* value <0.01, ^*∗∗∗*^*p* value <0.001; Wald chi-square test.

**Table 3:** Prevalence and prevalence ratio of iron deficiency in nonpregnant and pregnant women stratified by age group and pregnancy trimester.

Group	ID			
	*N*	*n*	Prevalence, % (95% CI)	PR (95% CI)
Total				
NPW in fertile age^a^	3500	2553	72.9 (71.4–74.4)	1 (reference)
Pregnant women	1125	1066	94.8 (93.3–96.0)	1.30 (1.27–1.33)^*∗∗∗*^
1st trimester	41	36	87.8 (73.8–95.9)	1.20 (1.07–1.35)^*∗∗*^
2nd trimester	633	588	92.9 (90.6–94.8)	1.27 (1.24–1.31)^*∗∗∗*^
3rd trimester	451	442	98.0 (96.2–99.1)	1.34 (1.31–1.38)^*∗∗∗*^

18–26 years		3		
NPW in fertile age^a^	870	679	78.0 (75.1–80.8)	1 (reference)
Pregnant women	203	195	96.1 (92.4–98.3)	1.23 (1.18–1.29)^*∗∗∗*^
1st trimester	10	10	100 (69.2–100)	1.28 (1.24–1.33)^*∗∗∗*^
2nd trimester	117	110	94.0 (88.1–97.6)	1.21(1.14–1.28)^*∗∗∗*^
3rd trimester	76	75	98.7 (92.9–100)	1.26 (1.21–1.32)^*∗∗∗*^

27–35 years				
NPW in fertile age^a^	1285	932	72.5 (70.0–75.0)	1 (reference)
Pregnant women	723	682	94.3 (92.4–95.9)	1.30 (1.25–1.35)^*∗∗∗*^
1st trimester	23	19	82.6 (61.2–95.0)	1.14 (0.94–1.38)
2nd trimester	414	383	92.5 (89.5–94.9)	1.28 (1.22–1.33)^*∗∗∗*^
3rd trimester	286	280	97.9 (95.5–99.2)	1.35 (1.30–1.40)^*∗∗∗*^

36–44 years				
NPW in fertile age^a^	1345	942	70.0 (67.5–72.5)	1 (reference)
Pregnant women	199	189	95.0 (91.0–97.6)	1.36 (1.30–1.42)^*∗∗∗*^
1st trimester	8	7	87.5 (47.3–99.7)	1.25 (0.96–1.63)
2nd trimester	102	95	93.1 (86.4–97.2)	1.33 (1.25–1.42)^*∗∗∗*^
3rd trimester	89	87	97.8 (92.1–99.7)	1.40 (1.33–1.46)^*∗∗∗*^

CI; confidence interval; ID, iron deficiency; *N*, total number of participants; *n*, number of participants with iron deficiency; NPW, nonpregnant women; PR, prevalence ratio. ^a^Subgroups of nonpregnant women matched to the same age groups of the pregnant women. ^*∗∗*^*p* value <0.01, ^*∗∗∗*^*p* value <0.001; Wald chi-square test. Nonpregnant women ≤51 years versus >51 years.

## Data Availability

The descriptive statistics data used to support the findings of this study are included in the article. The filled forms used to support the findings of this study are available from the corresponding author upon request.

## Supplementary Materials

Supplementary Materials Supplementary Figure S1: sample form of the survey on anemia and iron deficiency applied during the screening conducted in mainland Portugal between 2013 and 2017 (English translation of the original Portuguese form).
