# Supplementary material for: Screening for Anemia and Iron Deficiency in the Adult Portuguese Population
Source: Anemia. 2020 Jul 29;2020:1048283. doi: 10.1155/2020/1048283 (PMC7411453; doi:10.1155/2020/1048283)
Supplement: Supplementary Materials — Supplementary Figure S1: sample form of the survey on anemia and iron deficiency applied during the screening conducted in mainland Portugal between 2013 and 2017 (English translation of the original Portuguese form). [file 1048283.f1.pdf]

Supplementary Materials

Supplementary Figure S1: Sample form of the survey on anemia and iron deficiency applied during the screening conducted in mainland Portugal between 2013-2017 (English translation of the original Portuguese form).

ANEMIA AND IRON DEFICIENCY – SURVEY

I. SOCIODEMOGRAPHIC CHARACTERIZATION

(1) **Age:**___

(2) **Gender**: **Male (M)** ♂ | **Female (F)** / Pregnant: No / **YES** ♀

(3) **Nationality:**___________

II. SYMPTOMS

(1) **Fatigue** in daily activities: No/ **YES** when
- **(SE)** Slight efforts (hygiene, walking on a flat floor)
- **(AE)** Average efforts (walking on a sloping floor)
- **(GE)** Great efforts (climb >3 flights of stairs)

(2) Bleed easily **(BE)**
Headaches **(HA)**
Dizziness **(DZ)**
No / **YES**

(3) **Visible blood loss**: No / **YES** in
- Feces **(Fc)**
- Urine **(Ur)**
- Nose / Mouth **(Ns/Mt)**
- Vagina (unrelated to menstrual bleeding) **(Vg)**
- Recent surgery (<3 months) **(Srg)**

III. PERSONAL BACKGOUND

(1) History of **ANEMIA**: No / **YES**, current therapy_______________

(2) **Chronic diseases:** No / **YES:**

Renal insufficiency – WITHOUT dialysis **(R)**	Heart failure **(HF)**
Renal insufficiency – dialysis **(RD)**	Coronary disease (angina, infarction) **(Cor)**
Gastritis **(G)**	Valvular heart disease (prosthesis) **(VH)**
Crohn's disease **(Crohn)**	Celiac disease **(Cel)**
Ulcerative colitis **(UC)**	
Intestinal polyps **(IP)**	Other (s) _________________

Supplementary Table S2: Prevalence and prevalence ratio of anemia and iron deficiency stratified by type of location of the participant.

Type of institution	n (%)	Anemia (N = 11030) Prevalence, % (95% CI)	Anemia (N = 11030) PR (95% CI)	ID (N = 11030) Prevalence, % (95% CI)	ID (N = 11030) PR (95% CI)
Health institutions	4653 (42.2)	56.7 (55.2-58.1)	1.59 (1.50-1.68)***	59.6 (58.2-61.0)	1.04 (0.99-1.10)
Public	2173 (19.7)				
Private	2480 (22.5)				
Other public locations	6377 (57.8)	35.6 (34.5-36.8)	1 [Reference]	57.0 (55.8-58.3)	1 [Reference]
Medical and health congresses	1544 (14.0)				
Companies	1106 (10.0)				
Pharmacies and Shopping Centers	3727 (33.8)				

Abbreviations: CI; confidence interval; ID, iron deficiency; N, total number of participants; PR, prevalence ratio.

***P-value <0.001; Wald chi-square test.
